# Autoimmune Limbic Encephalitis With Leucine-Rich Glioma-Inactivated 1 (LGI1) Antibodies Presenting as Rapidly Progressive Cognitive Decline and Psychiatric Symptoms: A Case Report

**DOI:** 10.7759/cureus.105211

**Published:** 2026-03-14

**Authors:** Jehan Zeb, Sadia Shoaib, Rifat Alam, Saddam Abbasi, Naheeda Bibi, Perinpanathan Niranjini

**Affiliations:** 1 Internal Medicine, Luton and Dunstable Hospital, Luton, GBR; 2 General Medicine, Luton and Dunstable Hospital, Luton, GBR; 3 Geriatrics, Luton and Dunstable Hospital, Luton, GBR

**Keywords:** autoimmune limbic encephalitis, hyponatraemia, immunotherapy, lgi1 antibody, psychiatric symptoms, rapidly progressive dementia

## Abstract

Autoimmune limbic encephalitis (LE) with leucine-rich glioma-inactivated 1 (LGI1) antibodies is rare but treatable, often presenting as rapidly progressive cognitive decline, behavioural changes, and hyponatraemia. These features can resemble psychiatric or neurodegenerative disorders, delaying diagnosis. We report a case of a 63-year-old man with confusion, memory loss, dream enactment, hallucinations, severe hyponatraemia, and specific higher cortical features, including apraxia, anomia, and executive dysfunction. Faciobrachial dystonic seizures (FBDS), a recognised hallmark of LGI1 antibody-mediated LE, were not clearly appreciated at initial presentation in this case. MRI revealed bilateral medial temporal lobe abnormalities, while CSF and EEG results were unremarkable. LGI1 antibodies were identified only after initial neuronal antibody tests were negative. Treatment with corticosteroids and intravenous immunoglobulin (IVIg) stabilised his condition and led to partial improvement. This case underscores the diagnostic challenges of LGI1 antibody-associated LE and the importance of early recognition, antibody testing, and prompt immunotherapy to prevent irreversible neurological damage. The objective of this case report is to highlight the diagnostic pitfalls and management considerations in LGI1 antibody-mediated LE presenting predominantly with psychiatric and cognitive symptoms.

## Introduction

Autoimmune limbic encephalitis (LE) is an inflammatory disorder of the medial temporal lobes characterised by rapidly progressive cognitive decline, behavioural changes, psychiatric symptoms, and seizures. It may occur as a paraneoplastic condition or as a primary autoimmune process mediated by antibodies against neuronal cell-surface or intracellular antigens. Importantly, unlike T-cell-mediated paraneoplastic forms, antibody-mediated LE targeting neuronal surface antigens is often reversible with timely immunotherapy [1].

Leucine-rich glioma-inactivated 1 (LGI1) antibody-associated LE is a well-recognised subtype. LGI1 is a neuronal protein involved in synaptic transmission and the regulation of excitatory signalling within the hippocampus. Autoantibodies against LGI1 disrupt this signalling, leading to limbic dysfunction manifesting as memory impairment, psychiatric symptoms, seizures (particularly faciobrachial dystonic seizures (FBDS)), sleep disturbances, and hyponatraemia [2,3]. It predominantly affects middle-aged and older adults and represents one of the more common non-paraneoplastic autoimmune encephalitides, although it remains relatively uncommon in routine clinical practice [1,3].

Autoimmune LE should be suspected in patients with rapidly progressive cognitive impairment, behavioural changes, or atypical seizures. However, significant clinical overlap with psychiatric disorders, neurodegenerative dementias, and prion diseases often leads to misdiagnosis and delayed treatment. Unlike these conditions, LGI1 antibody-mediated LE typically responds well to immunotherapy, and early intervention is associated with better cognitive outcomes and reduced long-term sequelae [1].

This case report describes a patient with LGI1-positive LE who was initially misdiagnosed with dementia and psychiatric illness, resulting in delayed immunotherapy. By highlighting the diagnostic challenges and consequences of delayed recognition, this report reinforces the importance of early antibody testing and increased clinical awareness in patients with rapidly evolving cognitive syndromes.

## Case presentation

A 63-year-old man with asthma and gastroesophageal reflux, who had previously been cognitively intact, fully independent, and working without difficulty, developed subacute involuntary movements, confusion, insomnia, dream enactment, and worsening cognitive and behavioural symptoms, including mutism, anomia, visual hallucinations, irritability, and severe memory loss. Family reported frequent nocturnal falls from bed, consistent with parasomnia. On retrospective collateral history obtained from family members, the patient was also noted to have frequent episodic facial twitching and abnormal movements involving the left upper limb during the early weeks of his illness. These episodes were brief and intermittent.

During the initial admission, the patient was managed in a side room due to concern for infective encephalitis, limiting continuous direct observation by clinical staff. Additionally, he was under psychiatric care for agitation and intermittent aggressive behaviour. He was treated with antipsychotics and, as needed, sedative medications, which may have masked the typical semiology of FBDS. As a result, classical FBDS were not clearly recognised at presentation.

Examination revealed subtle cortical signs, including ideomotor apraxia, use of body parts as tools, and executive dysfunction. He could not identify the date or perform reverse sequencing tasks, but remained oriented to place and person. He had persistent, severe hyponatraemia with a sodium level of 115 mmol/L (Table 1). Further evaluation in conjunction with the endocrinology team demonstrated findings consistent with the syndrome of inappropriate antidiuretic hormone secretion (SIADH), a recognised association of LGI1 antibody-mediated LE [4]. All other routine blood tests, including complete blood count, renal and liver function, thyroid function, vitamin B12 and folate, inflammatory markers, and HIV serology, were normal. Autoimmune screening was negative (Table 1). CSF analysis showed no evidence of infection, malignancy, or inflammation. EEG recordings demonstrated only nonspecific slow-wave activity, a finding common in LGI1 antibody-mediated LE, and did not exclude the diagnosis [5]. A whole-body PET scan and CT thorax, abdomen, and pelvis were performed and showed no evidence of underlying malignancy.

**Table 1:** Laboratory test results

Test	Results	Reference Value
C-reactive protein	<0.3 mg/L	0-4.9 mg/L
White blood cell count	6.2 × 10^9^/L	4-11 × 10^9^/L
Haemoglobin	140 g/L	130-165 g/L
Platelets	265 × 10^9^/L	150-450 × 10^9^/L
Sodium	115 mmol/L	133-146 mmol/L
Potassium	4.8 mmol/L	3.5-5.3 mmol/L
Creatinine	70 µmol/L	62-106 µmol/L
Urea	4.7 mmol/L	2.5-7.8 mmol/L
Vitamin B12	424 pg/mL	191-663 pg/mL
Folate	6.5 µg/L	4.5-32.2 µg/L
Free thyroxine	19.6 pmol/L	11-22 pmol/L
Thyroid-stimulating hormone	1.42 mIU/L	0.27-4.2 mIU/L
Cortisol	446nmol/L	133-537 nmol/L
Urine osmolality	540 mOsmol/kg	50-1200 mOsml/kg
Serum osmolality	254 mOsmol/kg	275-295 mOsml/kg
Urine sodium	43 mmol/L	>20 mmol/L
Urine potassium	42 mmol/L	25-125 mmol/L

**Figure 1:**
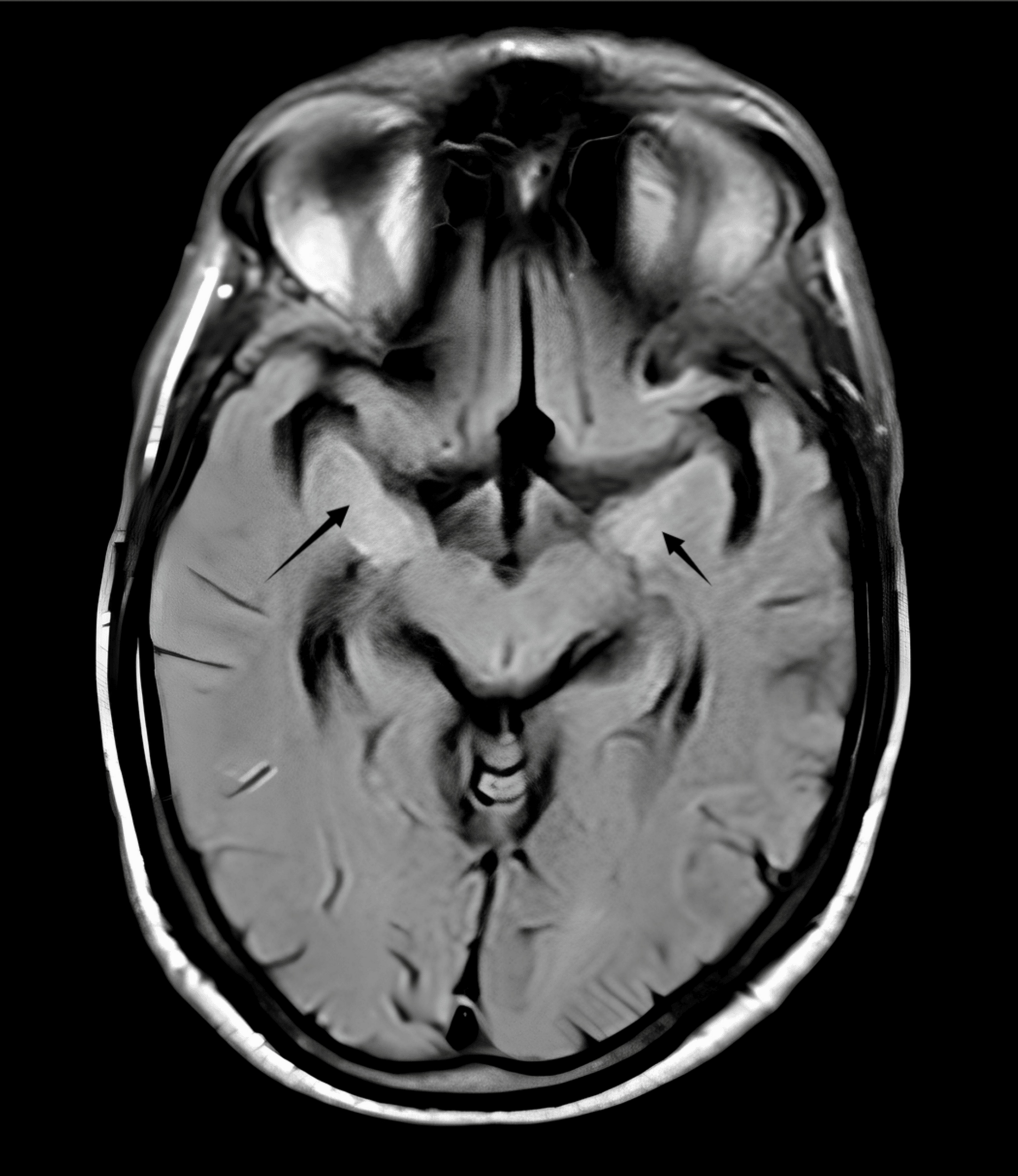
Brain MRI (T2/FLAIR SPIR) showing bilateral medial temporal lobe hyperintensities (arrows) FLAIR, fluid-attenuated inversion recovery; SPIR, spectral presaturation with inversion recovery

Brain MRI demonstrated bilateral medial temporal lobe hyperintensities, more pronounced on the right, with involvement of the insular cortex, as shown in Figure 1. There was no evidence of diffusion restriction, haemorrhage, or mass effect. These findings were consistent with inflammatory involvement of the limbic system, supporting the diagnosis of LE [6].

A comprehensive neuronal antibody panel was performed. Most results were negative and available promptly, but the LGI1 antibody result was delayed and later returned positive (Table 2) [7]. The clinical presentation, MRI findings, and LGI1 antibody positivity confirmed the diagnosis of LGI1 antibody-mediated LE.

**Table 2:** Neuronal antibodies panel LGI1, leucine-rich glioma-inactivated 1; CASPR2, contactin-associated protein-like 2; NMDA, N-methyl-D-aspartate

Test	Result	Normal Range
LGI1 antibodies	Positive	Negative
CASPR2 antibodies	Negative	Negative
NMDA receptor antibodies	Negative	Negative
Neuronal nuclear antibody 1 (HU)	Negative	Negative
Neuronal nuclear antibody 2 (RI)	Negative	Negative

The patient received a five-day course of high-dose intravenous methylprednisolone, followed by a tapered course of oral prednisolone. After initial stabilisation, he was discharged on oral steroids. However, he developed worsening psychiatric symptoms, including agitation, mood lability, hallucinations, and suicidal ideation, raising concern for steroid-induced effects. He was readmitted, corticosteroids were reduced, and intravenous immunoglobulin (IVIg) (2 g/kg over five days) was administered.

After immunoglobulin therapy and the addition of mirtazapine and melatonin, psychiatric symptoms partially improved. He was discharged after completing IVIg therapy, with outpatient follow-up arranged. At the time of discharge, he was mobilising independently and largely independent in activities of daily living; however, he remained intermittently confused, required occasional support from family members, and had not yet returned to work. At follow-up, the patient’s abnormal involuntary movements had resolved; however, he continued to experience low mood and persistent anterograde memory impairment and remains under ongoing neurology and psychiatry review.

## Discussion

This case highlights the diagnostic challenges of autoimmune LE with LGI1 antibodies, which may present with neuropsychiatric symptoms and unexplained hyponatraemia, mimicking psychiatric or neurodegenerative conditions [4]. MRI evidence of bilateral medial temporal lobe hyperintensities, with normal EEG and CSF findings, was critical for early diagnosis [6]. MRI should be prioritised in suspected cases when other investigations are inconclusive [5].

Similar cases of LGI1 antibody-mediated LE presenting predominantly with psychiatric symptoms and initially misdiagnosed as primary psychiatric illness have been described in the literature, further underscoring the diagnostic complexity of this condition [8].

LGI1 antibodies target components of the voltage-gated potassium channel (VGKC) complex at synaptic sites, disrupting synaptic transmission and leading to neuronal hyperexcitability within limbic circuits. This mechanism underlies the characteristic combination of seizures, cognitive impairment, and behavioural disturbance seen in LGI1 antibody-mediated LE.

FBDS are considered a key and often pathognomonic clinical feature of LGI1 antibody-mediated LE. However, FBDS may be subtle, intermittent, or masked in patients with prominent psychiatric symptoms, limited clinical observation, or exposure to sedative or antipsychotic medications. In this case, FBDS were not initially appreciated, but retrospective collateral history from family members suggested episodic facial and upper limb movements consistent with FBDS. This highlights the importance of obtaining detailed collateral history and maintaining a high index of suspicion when evaluating patients with rapidly progressive cognitive and behavioural decline.

Antibody testing confirmed the diagnosis, though the delayed LGI1 result highlights a common clinical challenge. Treatment is often started based on clinical judgment and imaging. LGI1 antibody-mediated LE is typically responsive to immunotherapy, particularly corticosteroids and IVIg, with improved outcomes when treatment is initiated early [7]. In this case, high-dose corticosteroids led to partial improvement but caused psychiatric side effects. IVIg was better tolerated and stabilised symptoms. Imaging excluded malignancy, consistent with the lower cancer risk in LGI1 encephalitis [7].

Relapse has been reported in up to approximately 30% of patients with LGI1 antibody-mediated LE, and increasing evidence supports the use of longer-term immunosuppressive strategies to reduce recurrence [9-11]. In relapsing or refractory cases, second-line therapies such as rituximab or cyclophosphamide are commonly employed, while steroid-sparing agents, including azathioprine, may be considered for maintenance immunosuppression. Given the evolving understanding of disease course and relapse prevention in LGI1 encephalitis, close long-term clinical monitoring and individualised decisions regarding the duration of immunosuppression are recommended. Clinical improvement in LGI1 antibody-mediated LE often precedes serological antibody decline, and serial measurement of antibody titers is therefore not always necessary or reliable for assessing treatment response or clinical recovery.

Prompt recognition of autoimmune encephalitis in patients presenting with cognitive decline, behavioural changes, hallucinations, and hyponatraemia is essential [8]. Delayed diagnosis increases the risk of irreversible deficits [12]. Treatment should be initiated when clinical suspicion is high, even before antibody confirmation.

This case further emphasises the importance of collaboration between neurology and psychiatry in patients with overlapping neurological and psychiatric symptoms. Multidisciplinary management is essential for accurate diagnosis and effective treatment [13].

## Conclusions

LGI1 antibody-mediated autoimmune LE is a potentially reversible cause of rapidly progressive cognitive and psychiatric decline. Clinicians should maintain a high index of suspicion in patients presenting with new-onset memory impairment, behavioural changes, hallucinations, seizures, or unexplained hyponatraemia, even when EEG and CSF findings are nonspecific or normal. Neuroimaging findings such as bilateral medial temporal lobe T2/fluid-attenuated inversion recovery (FLAIR) hyperintensities may provide supportive evidence of limbic system inflammation but are not independently diagnostic and must be interpreted in conjunction with clinical features and antibody testing. Definitive diagnosis relies on the detection of LGI1 antibodies in the appropriate clinical context.

Our patient’s partial clinical improvement following initiation of immunotherapy highlights the importance of early treatment, even before antibody confirmation, as timely intervention is associated with improved neurological outcomes. Nevertheless, residual cognitive and neuropsychiatric deficits may persist despite initial recovery. Long-term neurological and psychiatric follow-up remains essential due to the risk of relapse and ongoing impairment. As extended follow-up data were limited in this case, continued monitoring is necessary to better define long-term prognosis and optimise management strategies.
